# Little Things in Medicine

**Published:** 1888-06

**Authors:** N. B. Herring

**Affiliations:** Wilson, N. C.


### LITTLE THINGS IN MEDICINE.

By Dr. N. B. Herring, Wilson, N. C

[Read at the December Meeting of the Wilson County Medical Society.]

If I may be allowed to express myself, I purpose in this paper to speak of the *agenda* of littleness in medical practice, *i.e.*, to call your attention to some very small, but very useful things, that you will not find in the books; things that will count for you, in the long run, with the people, and make for you the distinction between the expert and the bungler. The crumenal state of our climate renders it unhealthy for trained assistants, and, as a matter of fact, we cannot always ignore.

### THE PHYSICIAN AS NURSE.

Many physicians, and all the laity, imagine that no one can take medicine or drink water lying flat on the back. Such is not the case. With very ill patients pursue the following method, and teach it to the nurse: Go to the patient with the medicine, water, stimulant or food, whatever it be, and with the magnetism of kindness and authority tell him to be quiet. Slip your hand under the pillow and tilt his head forward the least bit. Tell him to open his mouth, and pour the potion in. He will swallow it before he knows what he has done. If he objects, assure him that he will succeed, that it is as easy to swallow lying on the back as it is sitting up, and he will be astonished and gratified to find that he has accomplished that which, to him, appeared impossible.

No patient can get too sick to take a bath when he needs it: Get the bath ready, take off the patient's clothing and lay him on a strong sheet. Lift him off the bed into the bath by means of the sheet as though it was a hammock. After the bath lift him back on the bed in the same manner. The physician should always superintend such baths, and your own ingenuity will supply needed details. A little common-sense and tact will often be of more service to yourself and your patients than a knowedge of the most complicated formula in chemical pharmacy. I have seen an old physician of renown try to administer a clyster of castor oil with a Davidson syringe. Of course it was a failure, as all such instruments are intended for thin fluids only. There was nothing else at hand but a glass syringe with a slack piston. He attempted to fill that with the oil, but could never succeed in getting it more than one-fourth full, on account of the air rushing in beside the piston. In such a case draw in all that your instrument will take, invert the syringe and push out the air. Repeat your maneuvres, and success will crown your efforts with a very defect.ve instrument.

THE HYPODERMIC SYRINGE

is another instrument nearly alway out of order, and the proper use of which is little understood by the profession. The almost universal plan of introducing the needle at an angle of 45° is not the best. Pinch up the muscle with the thumb and finger, and press the needle perpendicularly into the muscle from half inch deep to the whole length of the needle, according to the size of the muscle. It gives less pain, is easier done, and produces less irritation. The salts of mercury, especially, introduced just beneath the skin, almost invariably leave a red spot, which fades slowly and may cause a slough. I have seen sulphuric ether inserted just under the skin leave a purple spot for weeks. The needle for ordinary use should be the smallest. It makes a great difference with your lady patients.

NOSE BLEED

is an affection the worst treated of any in surgery. The directions given for plugging the posterior nares would cause an intelligent ecclesiastic to assent to the doctrine of "descent" as formulated by Darwin and amplified by Hæckel. To imitate blindly, without thought, is the office of an ape or an ignorant negro, but not the duty of an intelligent physician. As laid down in the books on surgerv, it is barbarous, painful, unscientific, and only accomplishes its object by indirect means. Washes, douches, astringents, etc., are inefficient, and the doctor who would apply Monsel's solution to the nasal fossæ ought to be indicted for mal-practice. Pressure, when it can be applied, is the proper remedy for hemorrhage, as you all know. This is the object of all plugs. When the front and back ends of the canal are plugged the middle portion fills with blood, and the pressure is exerted by the clot. If it could be done easily and without pain, it is not the best way. These plugs have to come away sooner or later, and to remove them is painful and tedious, and the very means are liable to set up hemorrhage afresh. You are all aware that the nasal fossæ are a couple of horizontal canals about four inches long, with great depth and no width. They are nearly filled up with the scroll work, not for ornament, but to multiply the surface of the smelling apparatus. This scroll work forms, or is formed by the turbinated bones, and the mucous membrane covering them is a net-work of blood-vessels and nerves, very sensitive and very subject to hemorrhage. The smoothest instrument passed into these cavities will cause an unpleasant sensation. How abominable, then, must be the instrument known as Bellocq's canula, plugs of sponge or cotton, with possible pyæmia and death! all to stop a little blood from the nose.

HOW TO DO IT.

Take a piece of tough, raw, fat, salt pork, cut it wedge shaped, four inches long, half inch thick and three-quarters of an inch wide. Force it into the bleeding cavity—*clear through into the pharynx*—and the work is done. It is antiseptic, painless, and never fails to stop the hemorhage. It is easily removed, and the lookers-on are often solicitous to know what you put on the meat to produce such marvellous results. A hint to the wise (quack) is sufficient.

DRY CUPPING

to the spine is too much neglected, especially amongst neurotic females. No instruments are needed. A coffee cup, a piece of paper and a match. It will save many a dose of morphine and chloral.

THE SOFT CATHETER

is much better to use with women than the hard ones. You can't possibly do injury with a soft ca heter.

TO WASH OUT THE FEMALE BLADDER, sit the woman on the edge of a chair, fill a Davidson syringe with the wash. *and be sure it is full;* introduce the rectal nozzle into the bladder and slowly, very slowly, pump in the fluid. When the bladder becomes intolerant it will flow out beside the nozzle of the syringe, and then you may pump it in faster.

TO GIVE SMALL POWDERS TO INFANTS, wet the tip of your fore-finger with your tongue, get the powder on your finger, wipe it on the baby's tongue and let it nurse. This will save strangling the child or wasting the medicine.

TO MOP OUT A CHILD'S THROAT, twist a wad of absorbent cotton on the end of a stick and let the mop be large and bushy; dip into the wash, and at the right moment press it one time against the back part of the child's throat. This is much better than camel-hair pencils, as the pressure squeezes the wash out of the mop and diffuses it upon every part of the inflamed surface without a repetition of the process.

THE FLANNEL JACKET.

When you have a case of chest cold, pneumonia or bronchitis in a child, make a close fitting flannel jacket to reach from the clavicle to the lower ribs; saturate this with a mildly stimulating liniment, and sew it tightly around the child's chest. It is worth more than all the blisters, mustard-p asters, cups, etc., in existence. I let my little patients wear them several weeks, and sometimes all winter, occasionally applying the liniment. About the best liniment I have used is composed of—

| | | |
|---|---|---|
| R. | Castor oil .................................. | 4 parts, |
| | Spts. camphor .............................. | 2 parts, |
| | Spts. turpentine ............................ | 1 part, |
| | Tinct. cantharides ......................... | 1 part. |

ONE OF THE DELUSIONS

of medical practice is the rubbing of quinine on the surface of the body to get its therapeutic effect. The skin refuses absolutely to allow quinine to enter the system through its pores. Try it critically, patiently and alone, and you will be convinced.—*North Carolina Medical Journal.*

[We copy the above as containing a number of valuable suggestions. We accord with the writer except on one or two points. We do not like his method of the perpendicular puncture with the hyperdermic needle. It will do in sciatica or other cases where a deep local impression is desired, but ordinarily we prefer the oblique puncture, pinching up the skin on the arm where it is thin and injecting the fluid immediately beneath it.

Again, we do not like the coffeecup for dry cupping; a qunine bottle is far better. Dip a piece of paper in alcohol, touch it to a candle, drop in the bottle and apply quickly to the part. In the absence of a quinine bottle a glass tumbler is better than a cup.

As to quinine inunction, we think the skin does absorb a portion of the quinine when rubbed on with lard, but not less than three times the ordinary internal dose is required. Quinine with ether, alcohol or whiskey rubbed upon the skin is more effectual, and used during the fever will lower the temperature.—ED. RECORD.

